# Cerebrovascular disease and neuroinflammation predict change in Alzheimer's disease plasma biomarkers in adults with Down Syndrome

**DOI:** 10.1002/alz70856_105529

**Published:** 2026-01-09

**Authors:** Natalie C. Edwards, Patrick J. Lao, Mohamad J. Alshikho, Juyoung Hahm, Batool M. Rizvi, Lisi Flores‐Aguilar, Melissa Petersen, Sid E. O'Bryant, Benjamin L Handen, Jose Gutierrez, Donna M. Wilcock, Elizabeth Head, Adam Brickman

**Affiliations:** ^1^ Taub Institute for Research on Alzheimer's Disease and the Aging Brain, New York, NY, USA; ^2^ Taub Institute for Research on Alzheimer's Disease and the Aging Brain, Vagelos College of Physicians and Surgeons, Columbia University, New York, NY, USA; ^3^ University of California, Davis, Davis, CA, USA; ^4^ University of California, Irvine, Irvine, CA, USA; ^5^ Institute for Translational Research, University of North Texas Health Science Center, Fort Worth, TX, USA; ^6^ University of Pittsburgh, Pittsburgh, PA, USA; ^7^ Department of Neurology, Vagelos College of Physicians and Surgeons, Columbia University, and the New York Presbyterian Hospital, New York, NY, USA; ^8^ Indiana University School of Medicine, Stark Neurosciences Research Institute, Department of Neurology, Indianapolis, IN, USA

## Abstract

**Background:**

Adults with Down syndrome (DS) develop Alzheimer's disease (AD) pathology by age 40, despite few vascular risk factors. MRI shows cerebrovascular disease (CVD) that precedes or begins contemporaneously with markers of AD pathology. We have found that vascular lesions observed on MRI interact with a marker of astrocytosis to promote tau pathology and neurodegeneration. However, it's unclear how CVD and astrocytosis interact with elevated Aβ to influence AD progression. We investigated whether markers of CVD and astrocytosis are linked to longitudinal changes in tau biomarkers and their interaction with Aβ levels.

**Method:**

We included 114 participants (mean age[SD]=45.1[6]) from the Alzheimer's Biomarkers Consortium–Down Syndrome with baseline Aβ PET imaging ([11C]PiB or [18F]florbetapir) and three follow‐up visits with MRI and plasma biomarker data. White matter hyperintensity (WMH) volumes were derived from T2‐weighted FLAIR MRI, and plasma biomarkers (GFAP, *p*‐tau181) were measured at baseline and follow‐ups. Centiloid values were derived from Aβ standard uptake value ratio values and standardized across a scale from 0 to 100. Linear mixed effects models first estimated whether baseline WMH volume was associated change in GFAP concentration and whether baseline GFAP was associated with change in *p*‐tau181 concentration. Separate models then tested if each baseline measure interacted with amyloid centiloid values. Models included site, age, sex/gender, and years from baseline as fixed effects and a random effect for participant intercept.

**Result:**

Baseline WMH volume predicted an increase in GFAP over time (β=0.22[0.12, 0.31], AIC=‐102.84), and baseline GFAP predicted an increase in *p*‐tau181 (β=0.36[0.24, 0.4], AIC=‐122.66). However, baseline GFAP did not predict WMH change (β=0.07[‐0.1, 0.14], AIC = ‐25.7) nor did *p*‐tau181 predict GFAP change (β=0.009[‐0.18, 0.09], AIC=‐33.41). Higher WMH volume predicted increased GFAP, especially in those with higher amyloid levels (β=0.38[0.26, 0.45], AIC=‐146.3), and higher GFAP predicted increased *p*‐tau181, particularly in those with higher amyloid levels (β=0.49[0.32, 0.55], AIC=‐154.86).

**Conclusion:**

Cerebrovascular lesions promote astrocyte‐related inflammation, particularly in the context of elevated amyloid pathology, which has a downstream effect on tau pathophysiology in adults with DS. The results support the hypothesis that the interface between CVD and astrocytosis is critical in AD progression in people with DS.

